# Construction of the third-generation *Zea mays* haplotype map

**DOI:** 10.1093/gigascience/gix134

**Published:** 2017-12-30

**Authors:** Robert Bukowski, Xiaosen Guo, Yanli Lu, Cheng Zou, Bing He, Zhengqin Rong, Bo Wang, Dawen Xu, Bicheng Yang, Chuanxiao Xie, Longjiang Fan, Shibin Gao, Xun Xu, Gengyun Zhang, Yingrui Li, Yinping Jiao, John F Doebley, Jeffrey Ross-Ibarra, Anne Lorant, Vince Buffalo, M Cinta Romay, Edward S Buckler, Doreen Ware, Jinsheng Lai, Qi Sun, Yunbi Xu

**Affiliations:** 1 Bioinformatics Facility, Institute of Biotechnology, Cornell University, Ithaca, NY, 14853, USA; 2 BGI-Shenzhen, Shenzhen 518083, China; 3 Department of Biology, University of Copenhagen, Ole Maaløes Vej 5, DK-2200 Copenhagen, Denmark; 4 Maize Research Institute, Sichuan Agricultural University, Wenjiang 611130, Sichuan, China; 5 Institute of Crop Science, Chinese Academy of Agricultural Sciences/National Key Facilities for Crop Gene Resource and Genetic Improvement, Beijing 100081, China; 6 Institute of Crop Science and Institute of Bioinformatics, Department of Agronomy, Zhejiang University, Hangzhou 310058, China; 7 Cold Spring Harbor Laboratory, Cold Spring Harbor, NY 11724, USA; 8 Department of Genetics, University of Wisconsin, Madison, WI 53706, USA; 9 Department of Plant Sciences, University of California, Davis, CA 95616, USA; 10 Institute for Genomic Diversity, Cornell University, Ithaca, NY 14853, USA; 11 US Department of Agriculture-Agricultural Research Service, Ithaca, NY 14853, USA; 12 International Maize and Wheat Improvement Center (CIMMYT), El Batan 56130, Texcoco, Mexico; 13 National Maize Improvement Center, China Agricultural University, Beijing 100193, China

**Keywords:** *Zea may*, sequencing, haplotype map, genotyping, variant discovery, linkage disequilibrium, identity by descent, imputation

## Abstract

**Background:**

Characterization of genetic variations in maize has been challenging, mainly due to deterioration of collinearity between individual genomes in the species. An international consortium of maize research groups combined resources to develop the maize haplotype version 3 (HapMap 3), built from whole-genome sequencing data from 1218 maize lines, covering predomestication and domesticated *Zea mays* varieties across the world.

**Results:**

A new computational pipeline was set up to process more than 12 trillion bp of sequencing data, and a set of population genetics filters was applied to identify more than 83 million variant sites.

**Conclusions:**

We identified polymorphisms in regions where collinearity is largely preserved in the maize species. However, the fact that the B73 genome used as the reference only represents a fraction of all haplotypes is still an important limiting factor.

## Background

Maize, one of the most important cereals in the world, also happens to be among the crop species with the most genetic diversity. Advances in next-generation sequencing technologies have made it possible to characterize genetic variations in maize at genomic scale. The previously released maize HapMap2 were constructed with the whole-genome sequencing data of 104 maize lines across predomestication and domesticated *Zea mays* varieties [1]. Since then, more maize lines have been sequenced by the international research community, and a consortium was formed to develop the next-generation haplotype map. The maize HapMap 3 consortium includes, among others, BGI-Shenzen, Chinese Academy of Agricultural Sciences, China Agricultural University (CAU), and International Maize and Wheat Improvement Center (CIMMYT). High-coverage data for 31 European and US Flint and Dent lines are also available in Unterseer et al. [2]. Altogether, in this work, we used a total of 1218 maize lines sequenced with depths varying from less than ×1 to ×59 [3].

A common approach in today's genetic diversity projects is to map the shotgun sequencing reads from each individual onto a common reference genome to identify DNA sequence variations, and the physical positions of the reference genome are used as a coordinate system for the polymorphic sites. A good example is the Human 1000 Genomes Project [4]. The computational data processing pipeline developed for the human project, GATK, has been widely adopted for identifying genetic variations in many other species [5].

As the sequencing technology is improved and sequencers’ base calling error model gets more accurate, the computational challenges in genotyping by short-read sequencing have shifted from modeling sequencer machine artifact errors to resolving genotyping errors derived from incorrect mapping of short reads to the reference genome. The problem is associated with the experimental design that uses the single-reference genome as a coordinate system. Taking maize as an example, the reference being used is a 2.1-Gb assembly from an elite inbred line B73 that represents 91% of the B73 genome [6] and was estimated to capture only ∼70% of the low-copy gene fraction of all inbred lines [7]. Sequence alignment software, however, can map 95%–98% of the whole-genome sequencing reads to the reference. That suggests that a high percentage of the reads were mapped incorrectly, either to the paralogous loci or highly repetitive regions underrepresented in the reference assembly. The genetic variants called from the mismapped reads need to be corrected computationally. The maize HapMap2 relied on linkage disequilibrium in the population to purge most of the bad markers caused by alignment errors. To construct maize HapMap 3, a new computational pipeline was developed from scratch to handle the sequencing data from 10 times more lines, and it also took advantage of the high-quality genetic map constructed from the GBS technology [7, 8], which was not present when HapMap2 was constructed.

Genome structure variation in the population, including transposition, deletion, duplication, and inversion of the genomic segments, poses another challenge in the HapMap projects. As the physical genomes of each of the individuals included in the HapMap projects vary both by size and structure, and there is no colinearity of all the sequence variants between the reference and genomes of each of the individuals, it is not always possible to anchor all genetic variants in a population onto a single reference coordinate system. As a compromise, markers included in the maize HapMap are defined as sites of the physical positions of the B73 alleles matching the markers’ consensus genetic mapped positions.

Here we present maize haplotype map version 3 (HapMap 3), which is a result of coordinated efforts of the international maize research community. The build includes 1218 lines and more than 83 million variant sites anchored to the B73 reference genome, version AGP v3.

## Data Description

The sequencing data used in this work are comprised of 12 497 billion base pairs in a total of 113 702 billion Illumina paired-end reads, originating from 1218 maize and teosinte lines [3]. The data were collected from several sources over several years and vary in quality, read length, and coverage. Basic information about various datasets and stages of the HapMap 3 project they were used in are summarized in Table 1. Each of the 1218 lines were sequenced at depths varying from less than ×1 to ×59, using reads of lengths ranging from 44 bp through 201 bp, averaging 110 bp. All reads were aligned to maize reference genome B73, version AGP v3, using BWA mem aligner [10]. Overall, 95%–98% of the reads were mapped to the reference genome, although only about 50%–60% with non-0 mapping quality.

**Table 1:** Sequence datasets used in various stages of HapMap 3

		Coverage per taxon					
Dataset	No. of taxa	Minimum	Maximum	Average	3.1.1	3.2.1unimp	3.2.1imp
HapMap2	103	1	18.5	4.1	+	+	+
Hapmap2 extra	44	4.2	42	11.5	+	+	+
CAU	725	0.06	36.8	1.75	+	+	+
CIMMYT/BGI	89	1.1	19	11	+	+	+
282–×2	271	0	9	2.2	-	+	+
282–×4	270	0.6	34.5	4.4	-	+	-
German [2]	31	8.3	59	17.4	-	+	+

Taxa from sets “HapMap2,” “HapMap2 extra,” and “CAU” partially overlap. The “282” libraries, sequenced twice, represent 271 taxa. A “+” means that the dataset was used in a given stage, “-” that it was not.

All sequence data used in this work are publicly available (see details below). Collection and publishing of this data do not violate any local or international legislation or guidelines.

## Analysis

### Initial variant discovery

**Figure 1:**
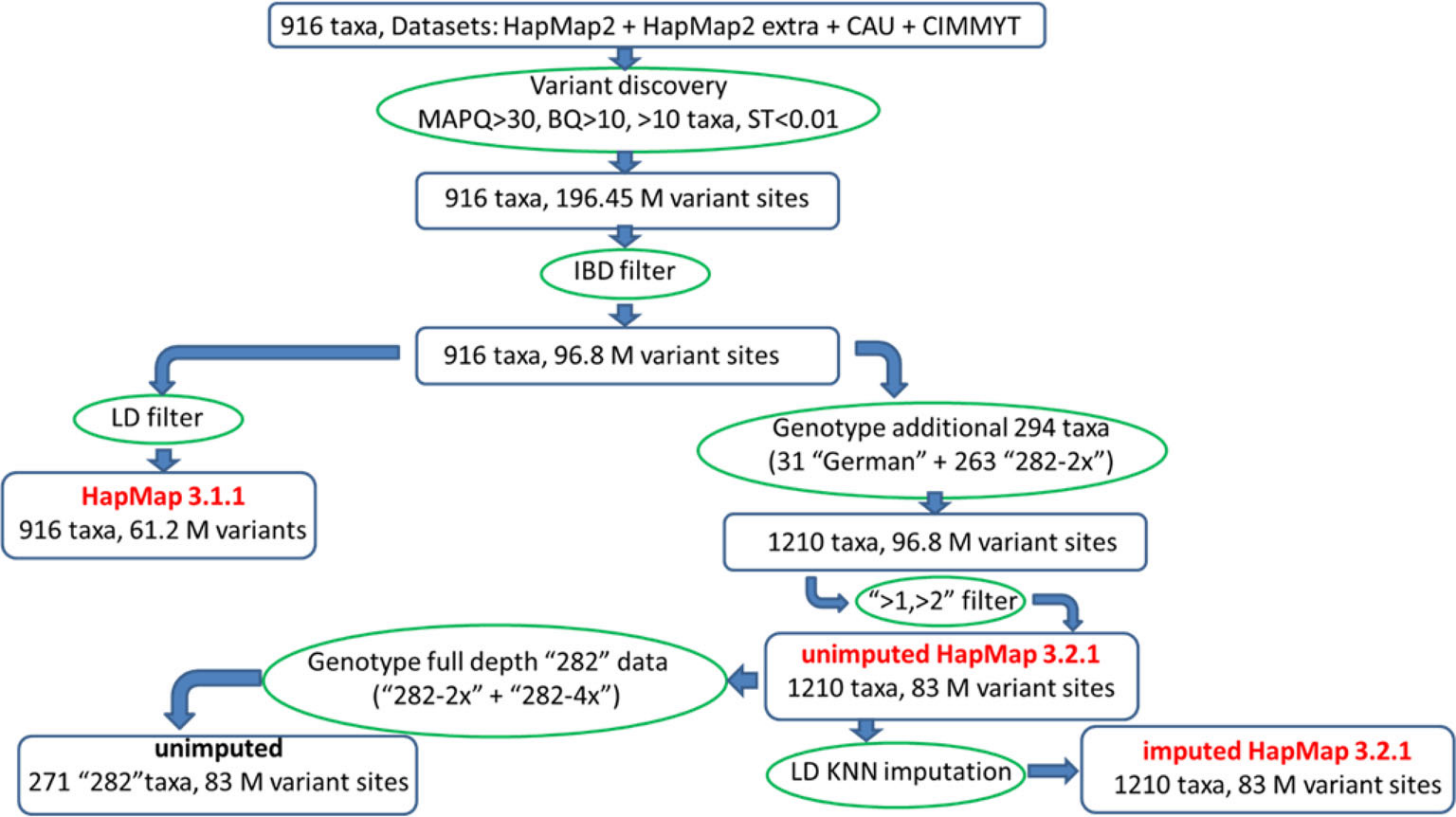
Overview of the HapMap 3 pipeline. Initial set of tentative variant sites was obtained from 916 taxa using reads with a mapping quality (MAPQ) of at least 30 and bases with a base quality of at least 10. At least 10 taxa had to have non-0 read coverage, and the *P*-value from the segregation test on allelic depths had to be at most 0.01. This initial set of sites was subject to filtering based on identity by descent. Application of a linkage disequilibrium filter eliminated sites with only nonlocal LD hits, leading to the HapMap 3.1.1 variant set. An alternative route, leading to HapMap 3.2.1 genotypes, involved K nearest neighbors imputation, in which distances were computed using sites in good local LD (hence, LD KNN). See the text for detailed explanation of methods and acronyms. The exact numbers of variant sites in HapMap 3.1.1 and HapMap 3.2.1 are 61 228 639 and 83 153 144, respectively.

The HapMap 3 pipeline is summarized in Fig. 1. First, polymorphic sites were called for a set of 916 taxa from datasets HapMap2 through CIMMYT/BGI (7191 billion base pairs, 74 643 million reads). In the first step, a custom-built software tool was used to determine genotypes for each taxon at each site of the genome based on allelic depths at that site. Bases that counted toward depth had base quality scores of at least 10 and originated from reads with mapping quality (MAPQ = − *int*(10*logP*), where *P*—calculated by the BWA mem aligner—is the probability of the reported read location being wrong) at or above 30. This cutoff was chosen at the midpoint between the highest MAPQ value reported by the aligner, corresponding to unambiguous alignments (60), and that of the most ambiguous ones (0). Analysis of the inbreeding coefficient (Section HapMap 3.1.1) and of MAPQ distributions shows that our choice of cutoff leads to decent quality genotypes while allowing for greater than 80% of alignments with MAPQ >0 to be included. Only sites where at least 10 taxa had coverage of 1 or more were considered. Following Unterseer et al. [2], at each site, the allelic read depths were subject to segregation test (ST; see the Methods section for details). For a population of inbred lines at true variant sites, one expects depths corresponding to minor and major alleles to be concentrated in roughly different subsets of taxa rather than being randomly distributed. The purpose of the ST test is to find and eliminate sites for which allelic depth distribution appears random, as such randomness, indicating high heterozygosity, is likely caused by alignment and sequencing errors. A measure of the randomness is the *P*-value of the ST test (the smaller the *P*-value, the less random the distribution). A *P*-value threshold of 0.01 was used in this study. This choice was somewhat arbitrary, aimed at reducing the number of tentative variant sites to a manageable size before further, more stringent filters were applied. In this first, ST-based round of filtering, a total of 196 million tentative polymorphic sites were selected. In the second step, these sites were filtered using the identity-by-descent (IBD) information derived from about 0.5 million high-quality polymorphisms obtained previously [9] using the genotyping-by-sequencing (GBS) approach [8]. These GBS variants (GBS anchor) were used to determine regions of IBD, where certain pairs of taxa are expected to have identical haplotypes. The tentative polymorphic sites violating these IBD constraints were then filtered out, leaving 96.8 million sites. At roughly half of the sites surviving this filter, the minor allele was not present in taxa involved in the tested IBD relationships. At such sites (typically with low minor allele frequencies), the satisfied IBD constraints do not confirm the existence of a variant. They are therefore less reliable and have been marked with an “IBD1” flag in the VCF files (see Table 2 for a summary of flags and parameters present in HapMap 3 VCF files). The ST- and IBD-filtered variant sites were then used in 2 separate procedures, leading to 2 versions of HapMap 3 genotypes, referred to as HapMap 3.1.1 and HapMap 3.2.1.

**Table 2:** Flags and parameters used in INFO field of VCF files in various HapMap 3 versions

Parameter	3.1.1	3.2.1 unimp	3.2.1 imp	Description
DP	+	+	+	Total read depth at the site
NZ	+	+	+	Number of taxa with called genotypes
AD	+	+	+	Allelic depths (reference, alternative in order listed in ALT field)
AC	+	+	+	Numbers of alternative alleles in order listed in ALT field
AQ	+	+	+	Average allele base qualities (reference, alternative in order listed in ALT field) computed in HapMap 3.1.1 from 916 taxa
GN	+	+	+	Numbers of genotypes (AA,AB,BB or AA,AB,AC,BB,BC,CC if 2 alt alleles present)
HT	+	+	+	Number of heterozygotes
EF	+	+	+	EF = het_frequency/(presence_frequency*minor_allele_frequency); computed in HapMap 3.1.1 from 916 taxa
PV	+	+	+	*P*-value from segregation test, computed in HapMap 3.1.1 from 916 taxa
MAF	+	+	+	Minor allele frequency (summed up over all alternative alleles)
MAF0	-	-	+	Minor allele frequency in unimputed HapMap 3.2.1
FH	+	-	-	Fraction of heterozygous taxa among the 506 taxa with more than 50% nonmissing genotypes on chr 10
FH2	+			Site with FH greater than 2%
IBD1	+	+	+	Only 1 allele present in IBD contrasts—based on 916 taxa of HapMap 3.1.1
LLD	+	+	+	Site in local LD with GBS map—based on 916 taxa of HapMap 3.1.1
NI5	+	+	+	Indel or site within 5 bp of a putative indel—from 916 taxa of HapMap 3.1.1
INHMP311	-	+	+	Site present in HapMap 3.1.1
ImpHomoAccuracy	-	-	+	Fraction of homozygotes imputed back into homozygotes
ImpMinorAccuracy	-	-	+	Fraction of minor allele homozygotes imputed back into minor allele homozygotes
DUP	-	-	+	Site with heterozygote frequency >3%—based on unimputed HapMap 3.2.1 genotypes

“+” and “-” indicate presence or absence, respectively, of a parameter or flag in a given version of HapMap. For example, “-++” means the parameter is present in the VCF file of both unimputed and imputed HapMap 3.2.1, and absent from HapMap 3.1.1. VCF files: Unless indicated otherwise, all parameters are computed from depths and genotypes in the current VCF file.

### HapMap 3.1.1

The HapMap 3.1.1 procedure involved checking for linkage disequilibrium of each site against the GBS anchor map [7, 8], which consists of markers located in hypo-methylated and genetically stable regions. Sites giving only very weak or only nonlocal (i.e., outside of 1-Mb radius) linkage disequilibrium (LD) hits were eliminated, which resulted in the final set of 61 228 639 polymorphisms. For slightly less than 40% of these sites, LD could not be conclusively calculated due to the small minor allele frequencies (MAFs), whereas the remaining sites, confirmed to be in local LD with the GBS anchor, have been marked with the flag “LLD.” Among the sites surviving all filtering steps, 8.7 million are indels or are located near (within 5 bp) an indel. These have been marked with the flag “NI5.” As a procedure to achieve consistent alignment across all reads covering the same indels—local realignment—is not computationally feasible at this scale and has not been performed, genotyping errors could occur, and, consequently, most such sites are tentative and should be treated with caution.

**Figure 2:**
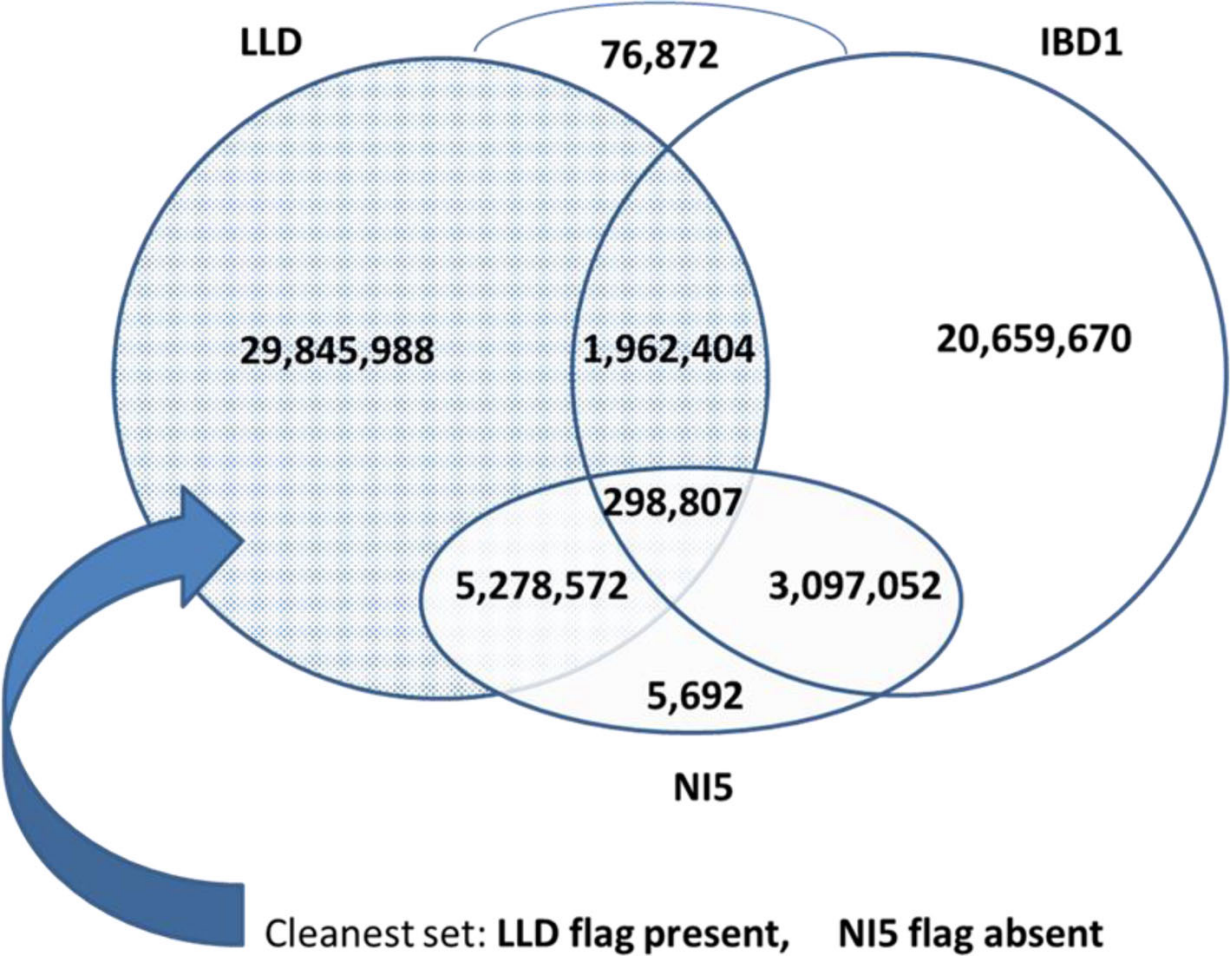
Overlap between various classes of HapMap 3.1.1 polymorphic sites. All sites listed passed the ST and IBD filters. LLD sites are those found in local LD with the GBS anchor. Sites flagged IBD1 passed the IBD filter; however, no alternative allele was present in IBD contrasts. Such sites do not violate IBD, but the existence of a variant is not confirmed. The NI5 flag is used to mark indels and sites within 5 bp of an indel. As no local re-alignment was done, the NI5 sites are not reliable.

Figure 2 shows overlaps between various classes of variants of HapMap 3.1.1. First, we notice a rather small overlap between sites in confirmed local LD (“LLD” flag) and those marked “IBD1.” This is understandable, as the IBD1 sites represent mostly low-MAF cases, where LD assessment could not be done. Indels and vicinity (labeled “NI5”) constitute about 15% of sites in each of the LLD, IBD1, and the union of LLD and IBD1 sets. Only a very small fraction of sites do not carry LLD or IBD1 flags; i.e., they are strongly confirmed by the IBD filter, but could not be classified with LD. The subset of 29.8 million sites in local LD and away from indels should be considered the most reliable.

**Figure 3:**
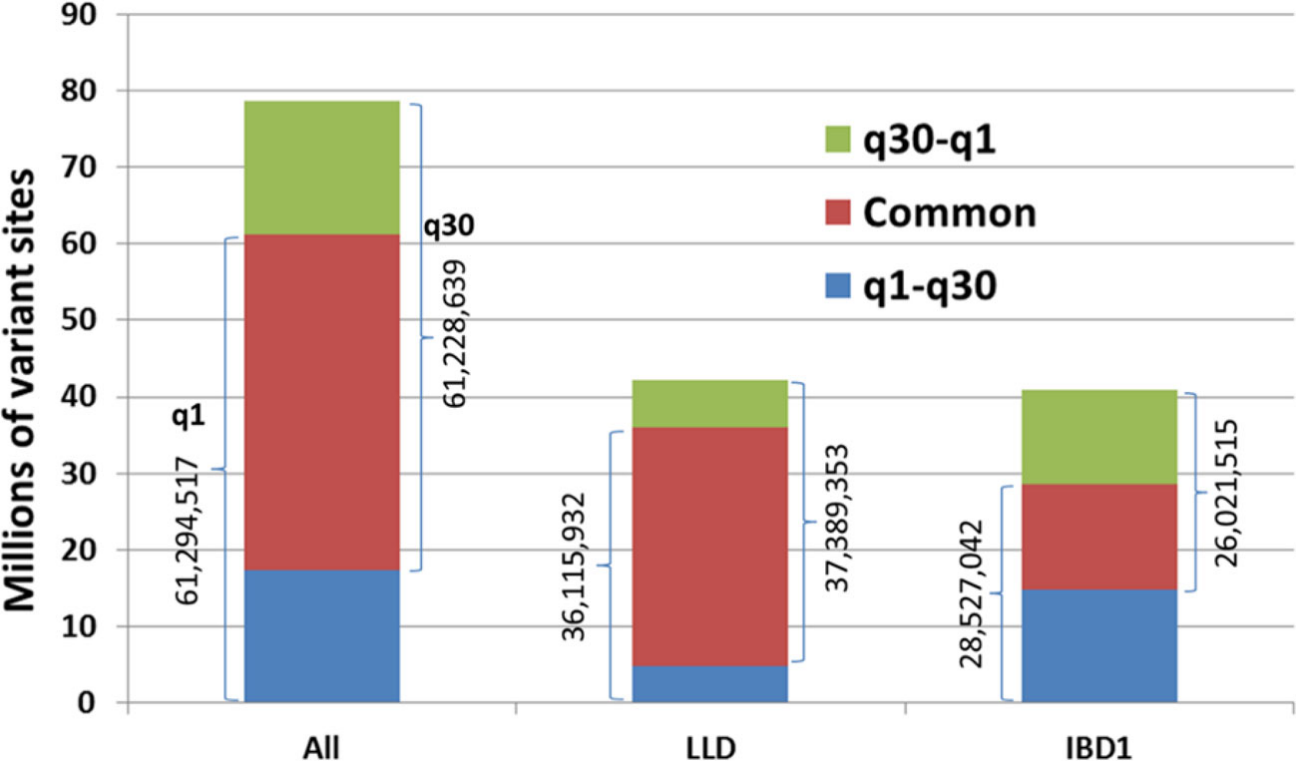
Polymorphic sites detected by the HapMap 3.1.1 pipeline based on 2 read mapping quality thresholds: MAPQ ≥1 (q1) and MAPQ ≥30 (q30). Tightening of the MAPQ threshold affects mostly the sites flagged with IBD1 (least reliable), while the LLD sites (in local LD with GBS anchor) are mostly independent.

To check the sensitivity of the obtained variant set to the mapping quality threshold imposed on the reads counted toward allelic depths, we repeated the pipeline using a mapping quality threshold equal to 1. Comparison of the variant set obtained this way (referred to as q1) with our recommended set (q30) is shown in Fig. 3. While the overall number of variant sites is approximately independent of the mapping quality threshold, the 2 pipelines produce significantly different sets of sites, with only 72% of all q30 sites reproduced by the q1 pipeline. Closer inspection shows that this variability is due primarily to the IBD1 sites, for which our filtering strategy was the least stringent. On the other hand, the LLD sites, confirmed to be in local LD with GBS anchor, are much more independent of the mapping quality threshold, which confirms the high quality of such sites.

For a population of inbred lines considered here, insight into genotype quality may be obtained from the inbreeding coefficient, calculated here for each taxon using the VCFtools program [12] from the formula

**Figure 4:**
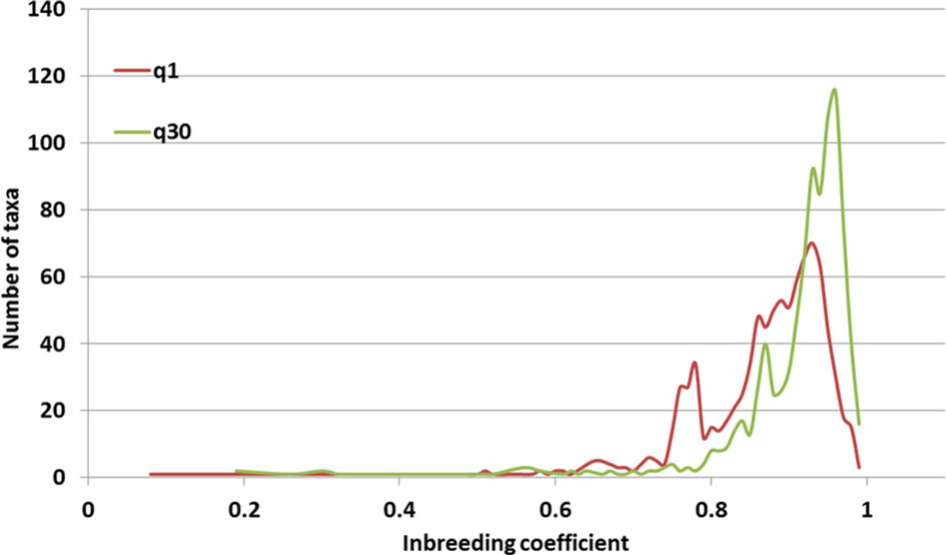
Distribution of inbreeding coefficient for HapMap 3.1.1 variant sets obtained with 2 read mapping quality thresholds: MAPQ ≥1 (q1) and MAPQ ≥30 (q30). A lower MAPQ threshold leads to lower values of inbreeding coefficient (i.e., higher heterozygosities) resulting from misaligned reads.

\begin{equation*}
\ {F_{inbr}} = \frac{{O - E}}{{N - E}},
\end{equation*}

where *O* is the observed number of homozygotes for a given taxon, *N* is the number of sites at which the taxon was genotyped, and *E* is the expected number of homozygotes given by $E\ = \mathop \sum \limits_k ( {1 - 2{p_k}{q_k}} )\ $. Summation in the latter formula runs over *N* genotyped sites, *p_k_* is the minor allele frequency at site *k* (computed from all taxa in the population with nonmissing genotypes at this site), and *q_k_* = (1 − *p_k_*). Low values of the inbreeding coefficient, indicating high heterozygosity, are mostly due to genotyping errors. The importance of choosing a sufficiently tight mapping quality threshold for the quality of genotypes is apparent from Fig. 4, where the distribution of the inbreeding coefficient for chromosome 10 is shown for the q1 and q30 variant sets. The lower MAPQ threshold results in a large number of mismapped reads being counted toward depth, producing overly heterozygous genotypes, especially for highly covered taxa (the peak below 0.8 is due mostly to CIMMYT lines with ×10–×15 coverage; these lines have higher heterozygosity than other lines, which may also contribute to the peak) and thus shifting the curve to the left. As most HapMap 3 taxa are inbred lines, one should expect the true distribution to be contained within peak around 0.95. In view of this, the q30 result is definitely an improvement over q1, although a longer-than-expected tail extending toward the value 0.8 indicates that the HapMap 3 variants may contain too many false heterozygotes.

Seemingly heterozygous sites may result from either sequencing errors or misalignments of reads originating from paralogous regions. To investigate this further, we calculated, for each site, the fraction of heterozygous HapMap 3.1.1 genotypes within a subset of 506 high-coverage taxa (defined as those with more than 50% nonmissing genotypes on chromosome 10). In HapMap 3.1.1 VCF files, this fraction has been recorded as parameter “FH.” At sites for which this parameter exceeds 2%–3%, heterozygotes are likely to originate from misalignments, e.g., from tandem and ectopic duplications. Such sites constitute 9% of all HapMap 3.1.1 sites.

### HapMap 3.2.1

The 96.8 million ST- and IBD-filtered variant sites were the starting point for the HapMap 3.2.1 procedure (Fig. 1). On these sites, genotypes were called on the 263 taxa from the “282” panel of Flint-Garcia et al. [11] using the “282–×2” dataset, and on the 31 high-coverage (on average ×17) “German” taxa [2], for a total of 1210 taxa. Some of the taxa present in the “282” and “German” sets carry the same names as the ones included in the 916-taxa HapMap 3.1.1 set. As despite identical names such taxa often originate from different germplasm sources, they have been kept separate during genotyping, i.e., reads from different sources were not merged, and separate genotypes were computed for each source. In the resulting VCF files, the names of the overlapping taxa have been prefixed by “282set_” and “german_.” For example, in the case of B73, there are 3 columns representing different datasets for this taxon: “B73” (the original 916-taxa set), “282set_B73” (sequence from the more recent “282” libraries), and “german_B73” [2].

To further eliminate the false positives resulting from sequencing errors, an additional depth-based filter was applied to the 96.8 million sites. Referred to as the “>1, >2” filter, it accepts sites for which the read support of minor alleles was greater than 1 in at least 1 taxon and greater than 2 across all taxa. Genotypes on the surviving 83 153 144 sites, referred to as “unimputed HapMap 3.2.1,” were then processed through the LD K nearest neighbors (KNN) imputation procedure based on Money et al. [13], where the “nearest neighbors” of a given line are selected based on sites in good local LD with the target site. Whenever possible, the procedure filled up missing genotypes with imputed ones, but the nonmissing genotypes were left unchanged, even if imputation classified them differently. Nonimputable missing genotypes at the sites with (pre-imputation) MAFs below 1% were assumed to be major allele homozygotes. Imputation reduces the fraction of missing genotypes from 50% to 7%. Most of the originally missing genotypes (about 85%) are imputed to major allele homozygotes. Accuracy of the genotype dataset can be assessed by comparing the original genotypes with imputed ones. As shown in Table 3, 99.8% of major allele homozygotes are imputed back into the same class. While the accuracies of minor allele homozygotes and genotypes including indels are both above 90%, only 11% of heterozygotes are imputed back into the same class, while 47% of them fail imputation altogether. This reflects the inherent difficulty in calling heterozygotes. In the single-reference approach to maize genotyping employed here, heterozygous sites represent true residual heterozygosity as well as misalignments of reads from tandem and ectopic duplications. As residual heterozygosity in our population of predominantly inbred lines should not exceed 2%–3%, all heterozygotes with frequency ≥3% can be considered a result of misalignments. About 10% of all heterozygotes present in the HapMap 3.2.1 set satisfy this condition. In the VCF files, these sites have been flagged with the flag “DUP” (“duplicated regions”). Other parameters generated by the imputation procedure and recorded for each variant site in the INFO field are ImpHomoAccuracy fraction of all homozygotes imputed back into homozygotes and ImpMinorAccuracy fraction of minor allele homozygotes imputed back to the same class. The INFO field also contains flags IBD1, LLD, and NI5, computed from the initial 916 taxa in the HapMap 3.1.1 procedure. Genotypes resulting from the imputation procedure are referred to as “imputed HapMap 3.2.1.”

**Table 3:** Accuracy of various genotype classes based on statistics from imputation in HapMap 3.2.1

Genotype class	Accuracy within class, %	% unimputed
Major allele homozygote	99.8	1.2
Heterozygote	11.1	47.0
Minor allele homozygote	94.4	14.2
Indel	92.2	17.3

Accuracy computed as percentage of the original number of genotypes in a given class (excluding genotypes that could not be imputed) imputed into the same class. The last column shows the fraction of genotypes within a class that could not be imputed.

**Figure 5:**
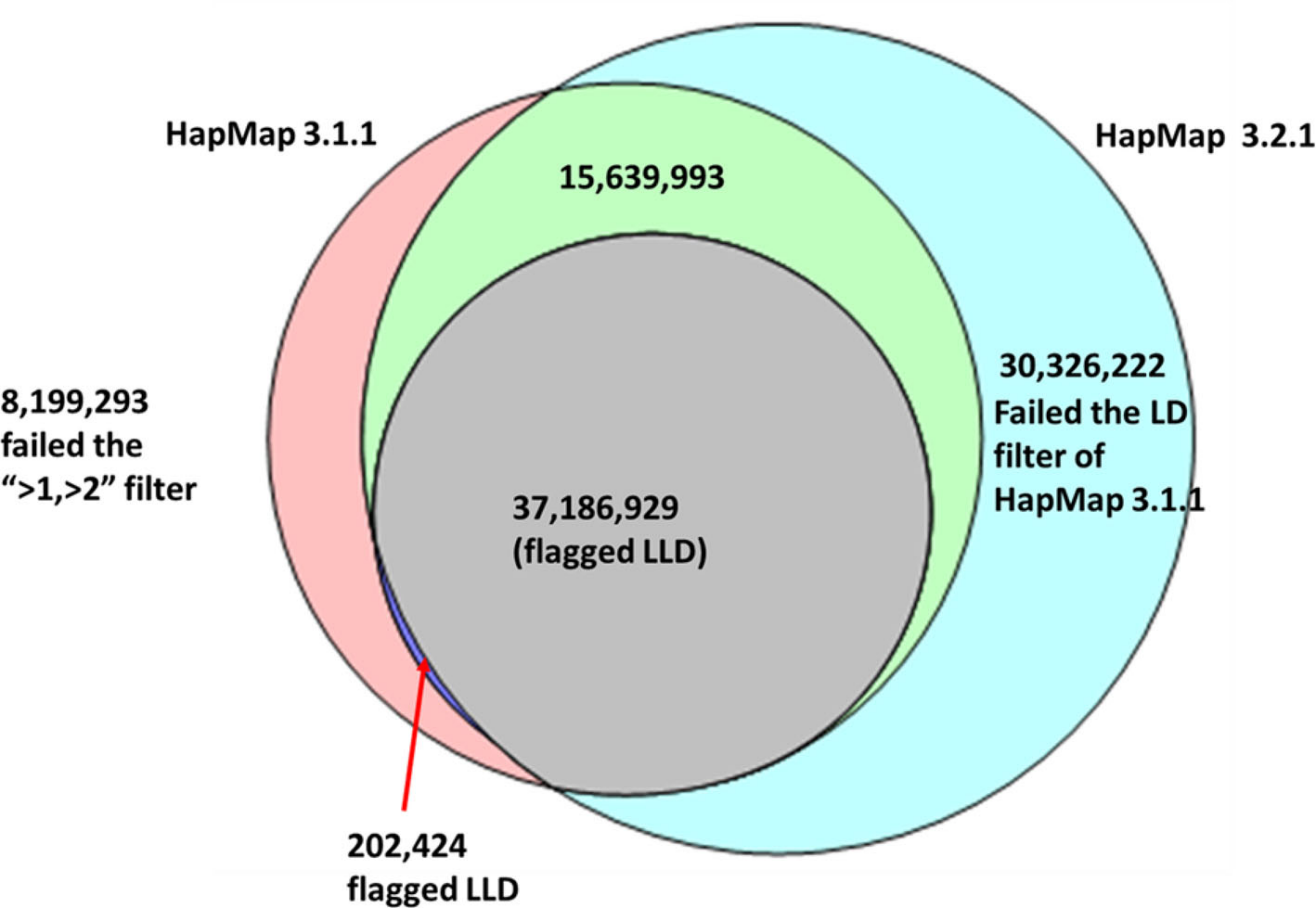
Overlap between HapMap 3.1.1 and HapMap 3.2.1 variant sites; 86% of HapMap 3.1.1 sites (99% of those in local LD) are recovered by the HapMap 3.2.1 pipeline.

The relationship between variant sites included in HapMap 3.1.1 and 3.2.1 is shown in Fig. 5. Both pipelines start from the same set of IBD-filtered genotypes and subject them to different kinds of filtering, with that of HapMap 3.1.1 being more stringent. It is therefore not surprising that HapMap 3.2.1 recovers the majority (86%) of HapMap 3.1.1 sites, including more than 99% of those flagged LLD (i.e., confirmed in local LD). In addition, 30.3 million extra sites are retained in HapMap 3.2.1, which failed the LD filer in the HapMap 3.1.1 pipeline. On the other hand, the depth-based “>1, >2” filter applied in HapMap 3.2.1 eliminated 8.2 million sites present in HapMap 3.1.1, including about 0.2 million LLD ones.

After the HapMap 3.2.1 release was completed, “282–×2” sequencing data became available for an additional 8 taxa from the “282” panel. Libraries for all 271 taxa were also resequenced at a higher depth (average of about ×4.4), leading to another dataset, “282–×4” (as this resequencing failed for 1 of the taxa, this dataset only contained 270 taxa). Therefore, the unimputed HapMap 3.2.1 genotypes for all 271 taxa from the “282” panel were recalled using the full available sequencing depth, creating a separate variant dataset for the “282” panel.

## Discussion

The maize genome, 2.3 GB in size [6], is smaller than the human genome. But some of its distinctive features make it more challenging for variant identification. First, a recent whole-genome duplication that occurred 12 million years ago resulted in homologous segments that complicate the short-read alignments; second, the rampant activities of transposable elements within last 1–5 million years not only resulted in the accumulation of large amounts of relatively young repetitive elements in the intergenic regions, but also extraordinary structural variations within species [5, 6]. In this study, the genome of the B73 maize line was used as the reference for variant calling from short sequencing reads. Structural variations between B73 and other individuals have been the major challenge for identification of true variants. In particular, short reads derived from regions missing in the reference genome could be mismapped to other paralogous regions, which could lead to false-positive genotypes. In the Human 1000 Genomes Project, a new HaplotypeCaller was used [5] that performs local *de novo* assembly to identify the most likely haplotypes for each individual and thus improve the genotyping results. However, HaplotypeCaller is computationally very expensive, and not always applicable in species like maize, where the single reference genome misses many haplotypes present in the species and has a lot more mismapped paralogous reads that would disrupt the local assembly. To filter out these false-positive variants called from the mismapped reads, we relied on the *Zea* GBS map [7, 8], which was obtained from GBS markers located primarily in hypo-methylated chromosomal regions. GBS maps were used to identify IBD regions between the individual genomes, and 100 million markers with a high percentage of mismatched genotype calls in the IBD regions were filtered out of the initial set of 196 million markers. The highly repetitive genomic regions derived from recent transposition activities are in general easier to identify, because the templates of these repeats are well represented on the reference genome, and sequencing reads mapped to these regions, flagged with low mapping quality, can be removed at an early stage of the analysis pipeline. For HapMap 3, reads with mapping quality lower than 30 were not included in the build.

One of the goals of HapMap 3.1.1 is to identify genetic markers in regions where collinearity is preserved in the majority of maize lines. The LD filter in the pipeline was applied for this purpose. To do this, we genetically mapped the presence/absence of the minor alleles using the GBS genetic map, and these mapped genetic positions were compared with the physical positions on the B73 reference. Among the 96.8 million sites surviving the IBD filter, 25% did not have enough nonmissing data or sufficient minor allele frequency for genetic mapping to be meaningful. For 38% of sites, at least 1 genetically mapped position matching the physical positions on the B73 reference was found, 24% had no significant hits from genetic mapping, probably due to no consensus positions in the HapMap 3 population, and 13% had genetic positions not matching the B73 physical positions. Markers from the latter 2 categories (37% of all IBD-filtered markers) were removed by the LD filter, leaving slightly over 61 million sites, about 60% of which were confirmed in local LD and marked with the flag “LLD” in VCF files.

The IBD and LD filters applied in the HapMap 3.1.1 project effectively remove the majority of the false-positive genetic variants caused by paralogous genomic regions, as well as markers with lost collinearity between the species. However, not all the genotyping errors have been removed from the release; 23 839 286 sites do not have sufficient minor allele frequency for genetic testing (these are missing the “LLD” label in the INFO field of the VCF files). Another source of errors is that paralogous regions evolved from tandem duplications. Misalignments of reads from such regions result in false heterozygous genotypes with relatively high frequency and in local LD, and are therefore difficult to filter out. Given enough sequencing depth, the tandem duplications can be identified either as copy number variations or imputation errors. However, the majority of the HapMap 3 lines have very low sequencing depth and fail to sample all paralogous loci or all alleles, which makes it difficult to flag all sites complicated by tandem duplications.

Local LD filter based on a large, diverse population may be too stringent, as some markers, good within certain subpopulations, may be thrown out. Therefore, the LD filter was not used in the HapMap 3.2.1 release, which contains a total of 83 million variant sites, subject only to ST and IBD filters and an additional depth-based filter aimed to improve the reliability of rare allele calls. Although those sites are likely to have higher misalignment rates, they are still likely to capture a true association with phenotypes.

**Figure 6:**
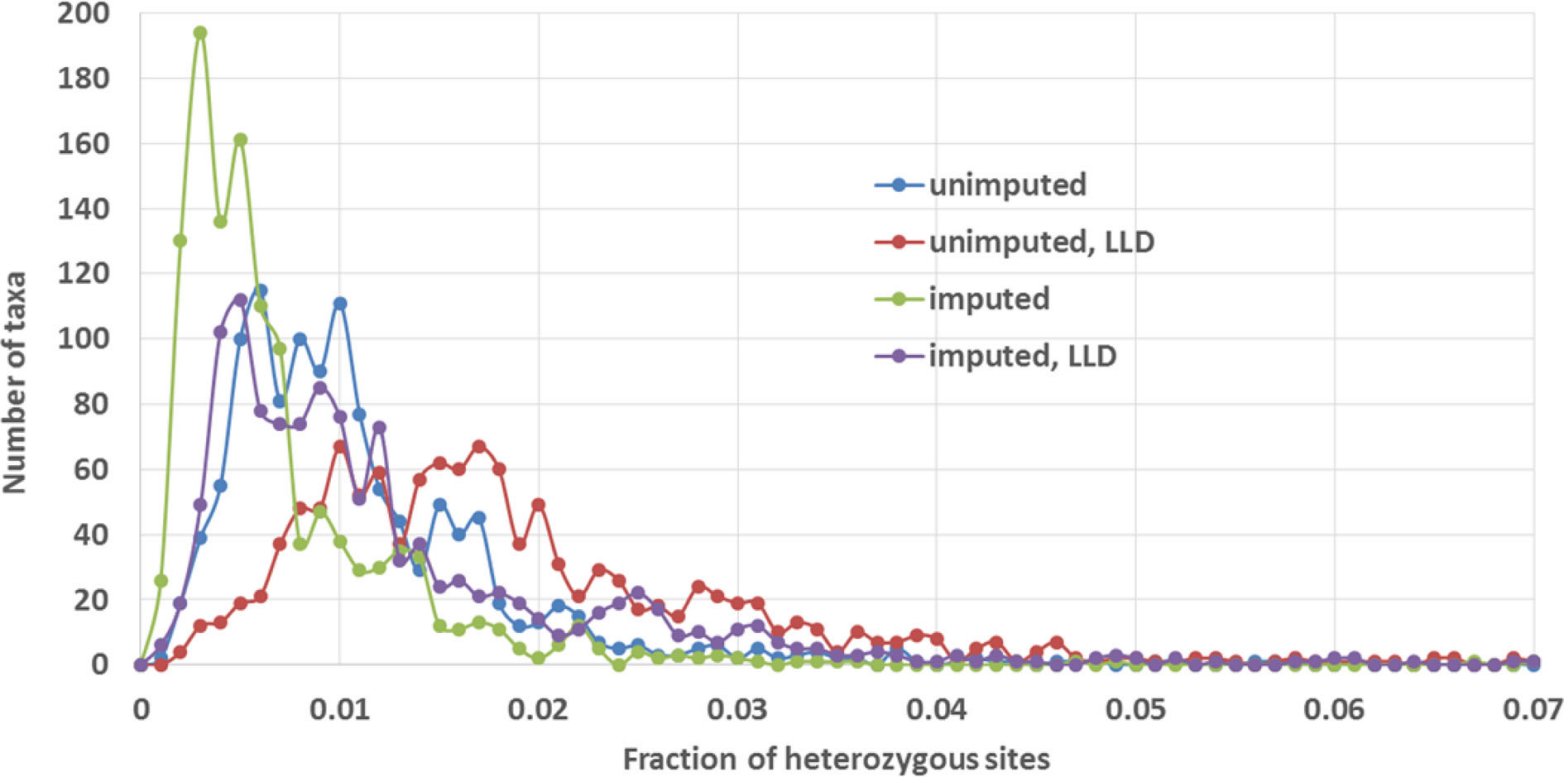
Distribution of fraction of heterozygous sites per taxon for unimputed and imputed HapMap 3.2.1. Curves marked LLD have been obtained considering only sites verified in HapMap 3.1.1 to be in good local LD with GBS anchor.

In the unimputed HapMap 3.2.1, at about 10% of all variant sites, a fraction of heterozygous taxa exceeds 3%. Such sites are marked “DUP,” as most likely originating from duplication misalignments. Figure 6 shows the distribution of the fraction of heterozygous sites per taxon for different versions of the HapMap 3.2.1 release. While for the unimputed genotypes the distribution peaks slightly below 1%, imputation significantly shifts the peak to the left, down to about 0.5%. This is a consequence of most missing genotypes being imputed to homozygotes. Interestingly, considering only sites in good local LD (marked with the “LLD” flag) leads to distributions (both imputed and unimputed) shifted toward higher heterozygosities. This is understandable, as the LLD sites are typically those with higher minor allele frequencies, where the chance of encountering a heterozygote is higher.

In summary, apart from the addition of more maize lines, the HapMap 3.2.1 release differs from the 3.1.1 release in 3 major aspects: (1) improved rare allele calls: to increase the accuracy of the variants with rare allele, the HapMap 3.2.1 pipeline applied more stringent read depth thresholds instead of the population genetics–based LD filter that could not be applied to sites with very low MAF; (2) the sites with a high percentage of heterozygous calls were flagged in the VCF files; (3) missing data were imputed using the LD KNN method. As summarized in Table 2, the VCF files of both datasets contain labels that flag the characteristics of each of the sites. To effectively use this resource, filtering the sites based on the flags that are appropriate to the purpose of each project is recommended.

When constructing the maize HapMap 3, the most serious problems we faced can be attributed to the use of a genome from a single individual (B73) as a reference for other, often very different species. This is becoming the single limiting factor in the study of maize diversity, as well as breeding practice. The only remedy is to move away from a single genome–based reference coordinate and adopt a pan-genome-based reference system that incorporates all major haplotypes of the species.

## Methods

### Plant material

Plant material used in this study was obtained mostly from maize inbred lines representing a wide range of *Zea mays* diversity; 103 of these lines, used previously in the HapMap2 project [1], include 60 improved lines, including the parents of the maize nested association mapping (NAM) population [14], 23 maize landraces, and 19 wild relatives (teosinte lines: 17 *Z. mays* ssp. *parviglumis* and 2 *Z. mays* ssp. *mexicana*). Sequence datasets originating from these lines are referred to in Table 1 as “HapMap2” and “HapMap2 extra.” The majority of the remaining inbred lines originated from CAU (sequence dataset “CAU”) and include, among others, “Chinese NAM” parent lines. An additional 89 inbred lines were provided by CIMMYT and sequenced at BGI (dataset “CIMMYT/BGI”). The HapMap 3 population also contained 1 *Tripsacum* line (TDD39103), 1 “mini-maize” line (MM-1A), and a few newly sequenced landraces. Overall, the number of taxa in the initial, variant-discovery stages of the HapMap 3.1.1 project was 916.

The sequence of 271 taxa from the libraries of the “282” panel [11] was added at a later stage (HapMap 3.2.1). DNA to construct these libraries was obtained from the collection that the North Central Regional Plant Introduction Station (NCRPIS) distributes all over the world. Additionally, the high-coverage data of Bukowski et al. [3], originating from 31 European and US inbreds, was also included. The total number of taxa genotyped in the HapMap 3.2.1 build is 1218.

In this study, individuals with the same taxa name but contributed by different members of the consortium were kept as separate entries in the genotyping pipeline—a decision prompted by comparison of genotypes obtained from different datasets. For example, the newly sequenced CML103 is significantly more heterozygous than CML103, studied previously in the HapMap2 project. Also, the Mo17 sequence originating at CAU has been treated as a taxon separate from Mo17 and CAUMo17. In most of those cases, a prefix or suffix indicating the origin of the sequence data has been added to the taxa name (e.g., “282set_” or “german_,” “-chin”).

### Sequencing

Sequencing has been performed over several years using various generations of Solexa/Illumina instruments and library preparation protocols, giving paired-end reads from 44 to 201 bp long. Overall, 113.7 billion reads were obtained on 1218 lines, containing 12 497 billion base pairs, giving on average ×4.4 coverage per line (assuming 2.3-Gb genome size). However, as shown in Table 1, coverage was not uniform among all lines. For a few lines, the sequence generated previously in the context of the HapMap2 project was augmented with reads from recent resequencing, which brought the median coverage of the HapMap2 lines to ×5, with an average coverage equal to ×7.8 and a standard deviation of ×7.2. All NAM parent lines are covered to ×10 or higher. Most of the 89 lines provided by CIMMYT and sequenced at BGI have coverage exceeding ×10. The recent resequencing of the “282” panel resulted in coverage between ×1.7 and ×36, averaging ×6.5. Coverage of the 31 “German” lines for Unterseer et al. [2] ranges from ×8.3 to ×59, with an average of ×17.4. The majority of the inbred lines that originated from CAU have been sequenced at a lower coverage (×1–×2). The list of all lines used in HapMap 3 with the corresponding coverage is given in Additional file 1.

### Alignment

**Figure 7:**
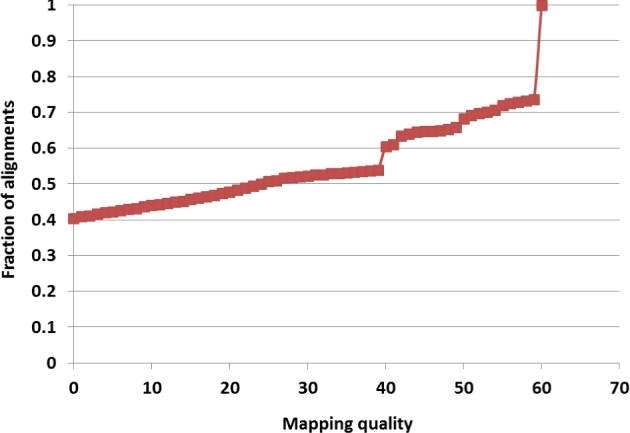
Cumulative distribution of mapping quality from BWA mem alignment of 125.4 million 150-bp reads from taxon A272.

Due to the use of different versions of Solexa/Illumina sequencing equipment, the base qualities in different input FASTQ files are given in different encodings. Prior to alignment, all base qualities have been converted to a phred+33 scale. Reads were then aligned to B73 reference (AGP v3) as paired-end using BWA mem aligner (1) with default options. In 72 read sets (Illumina lanes), for technical reasons, a high (6%–54%) fraction of paired-end fragments was found to be shorter than reads, so that the 2 ends contained a part of Illumina adapter and were reverse complements of each other. For such “read-through” fragments, the remnants of Illumina adapter sequences were clipped using TRIMMOMATIC (TRIMMOMATIC, RRID:SCR_011848) [15], and only 1 read was used and aligned as single-end. The BWA mem aligner is capable of clipping the ends of reads and splitting each read in an attempt to map its different parts to different locations on the reference. As a result, typically over 95% of reads are reported as mapped. However, the fraction of reads with non-0 mapping quality (negative log of the probability that a read has been placed in a wrong location) is much lower—typically only 40%–50%. Figure 7 shows a typical distribution of the mapping quality obtained from BWA mem alignment. In practice, we only used alignments with a mapping quality of at least 30. A base was counted toward allele depth if its base quality score was at least 10.

It is well known that alignment may be especially ambiguous when reads contain indels with respect to the reference. In such cases, multiple-sequence realignment approaches have been proposed [5] to find the correct sequence and location of an indel and avoid spurious flanking SNPs. Since indels are not the primary focus of this work and since the realignment is computationally very expensive, it has not been performed by the HapMap 3 pipeline. Thus, although indels and SNPs in their immediate vicinity have been retained in the HapMap 3 VCF files, they are less reliable and have therefore been marked with an “NI5” label for easy filtering.

### Genotyping pipeline

Raw genotypes were obtained using a custom-built multi-threaded java code [16]. First, the code executes the samtools mpileup command (thresholds on the base and mapping quality are imposed here) for each taxon individually, processing a certain portion of the genome. On a multi-core machine, several such pileup processes (i.e., for several taxa) can be run concurrently as separate threads. As we are predominantly interested in calling SNPs, we use a simplified indel representation where insertions and deletions with respect to the reference are treated as additional alleles “I” and “D,” respectively, regardless of the length and actual sequence of the indel. The read depths and average base qualities of all 6 alleles (A, C, G, T, I, and D) are extracted from samtools mpileup output for each taxon at each genomic position and stored in an array shared between all threads. The amount of memory available on the machine and the number of taxa determine the upper limit on the size of this array, and therefore the maximum size of chromosome chunk that can be processed at one time. As base quality of I and D alleles, we took the value corresponding to the base directly preceding the indel on the reference.

Extraction of allelic depths for all genomic positions is time-consuming, which presents a major obstacle if joint genotyping needs to be rerun, e.g., upon extending the taxa set. It is therefore advantageous to run the depth extraction only once for each taxon and save the obtained depths on the disk to be retrieved (rather than recalculated) during the genotyping step. This way, when the taxa set for genotyping is extended, the mpileup step has to be run only for the newly added taxa. Thus, the program features an option to save allelic depths and average qualities in specially designed data structures stored in HDF5 files—1 such file per taxon per chromosome. To save space, each allele depth and average quality is stored as 1 byte, which allows exact representation of integers from 0 to 182, while higher integers (up to about 10 000) are represented approximately by negative byte values through a logarithmic formula with a carefully chosen base. Depths and qualities are stored only for sites with non-0 coverage. The details of the storage format and integer representation in terms of byte variables are given in Additional file 2.

Once the allelic depths for all taxa and a given chunk of the genome are available in shared memory, each site is evaluated for the presence of a tentative SNP. On a multi-core machine, the set of sites within the genome chunk is divided into subsets processed in parallel on different cores. Sites with less than 10 taxa with read coverage and those with only reference alleles present are ignored. For all other sites, genotypes are called for all taxa using a simple likelihood model, with a uniform error rate [17] assumed at 1%. Alternative alleles are then sorted according to their allele frequencies, and up to 2 most abundant alleles are kept, as decided by the segregation test described in the next section. Sites for which all taxa turn out to be reference homozygotes (which may happen despite nonreference alleles being present in the mapped reads) are skipped. A raw variant set obtained in this way is then subject to extensive filtering, with the intention of reducing the number of false positives resulting from misalignments.

### Filtering

#### Segregation test filter

For each pair of alleles obtained in the genotyping step, a 2-by-N (where N is the number of taxa) contingency table is constructed, containing depths of the first allele in row 1 and the depths of the second allele in row 2. The Fisher exact test (FET) is then performed to assess how likely such a table is to occur by chance. If the expected values of the array elements are sufficiently large, the *P*-value from FET is approximated by that from the computationally efficient chi-square test. However, in most cases encountered here, expensive simulation is needed to obtain a sufficiently accurate *P*-value. To reduce computational burden, we adopted a hybrid approach based on an empirical observation that for statistically insignificant cases (*P*-values larger than 0.2), the chi-square test results in a *de facto* lower bound to exact *P*-values. Thus, the chi-square test is performed first for each site, and if the *P*-value from this test is below 0.2, a more exact *P*-value is obtained from a simulation procedure. The simulation procedure used here, implemented in Java, is the same as the one implemented in R package [18]. An alternative allele is kept if at least 1 contingency table involving this allele has a *P*-value smaller than or equal to 0.01. If none of the alternative alleles survive the ST filter, the site is skipped (not reported in output). The ST filter tends to eliminate variant sites resulting from random sequencing errors.

#### GBS anchor map and IBD filter

Given a set of trustworthy SNPs and a diverse set of 916 taxa, it is possible to identify, for an arbitrary region of the genome, the number of taxa pairs that are identical by descent and are therefore expected to have identical genotypes in this region. If known, these IBD pairs can be used as a powerful filter eliminating variants that violate IBD constraints.

To determine the IBD regions, we used the first step of our pipeline to call genotypes for our 916 taxa on the set of GBS v2.7 sites [7, 8], which tend to concentrate in relatively well-conserved low-copy regions of the genome and can therefore be considered reliable. This set of 954 384 sites was filtered to include only SNP (not indel) sites for which the *P*-value from the segregation test was below 0.05 and which were more than 5 bp away from any indel. The set of genotypes at 475 272 sites obtained in this way, which will be referred to as the GBS anchor, agree well with those from GBS on the 167 taxa present in both sets. Alleles detected by the HapMap 3 pipeline agreed with those from GBS at 94% of the GBS sites. At 90% of the sites, a fraction of (nonmissing data) taxa with genotypes in agreement with those from GBS was at or above 85%. Genotypes different from GBS ones were observed for 82 taxa. These differences were most frequent (up to 19% of all sites) for teosinte lines.

The GBS anchor was used to compute the genetic distance (identity by state) between any 2 of the 916 lines in windows containing 2000 GBS sites each (about 8.5 Mbp on average). If the genetic distance within such a window was ≤0.02 (about 10 times smaller than the mean distance across all pairs), the 2 lines were considered to be in IBD. At least 200 comparable GBS sites (i.e., nonmissing data simultaneously on both lines being compared) were assumed necessary to make the genetic distance calculation feasible. This allowed for a good distance estimate while keeping the number of detected IBD relationships large.

The number of taxa involved in IBD relationships in any given window was between 385 (start of chromosome 10) and 757 (middle of chromosome 7) and averaged 588, leading to large numbers of IBD contrasts, ranging from 3710 (beginning of chromosome 4) to 42 890 (middle of chromosome 7), and averaging 13 500.

The tentative (ST-filtered) variant sites were confronted with the IBD information as follows: For each site, pairs of lines in IBD were determined as described above. Genotypes of IBD-related lines were compared, and the numbers of allele matches and mismatches, summed over all IBD pairs, were counted for each allele present at the site. If the match/mismatch ratio was at least 2 for at least 2 alleles, or if only 1 allele was present in all IBD contrasts, the site was considered passing the IBD filter. Such a filter is less powerful for sites where all bases in IBD lines are major allele homozygotes, i.e., the variant being evaluated occurs in lines not involved in IBD pairs. Formally, such a site passes our IBD filter, but the actual variant is not strongly confirmed. These uncertain sites, mostly with low minor allele frequency, are labeled “IBD1” in the HapMap 3 VCF files and constitute about 50% of all HapMap 3 sites.

#### Linkage disequilibrium filter

Any true SNP should be in local linkage with other nearby SNPs. This observation is the origin of another filter used in this work, referred to as the LD filter. For each variable site surviving the ST and IBD filters, we evaluated the LD with each site of the GBS anchor. As the LD measure, we chose the *P*-value from a 2-by-2 contingency table of haplotype counts AB, Ab, aB, ab. For the purpose of counting haplotypes, heterozygous genotypes were treated as homozygous in minor allele, so that each taxon only contributed at most 1 haplotype. This tends to somewhat strengthen the LD signal and simplify the calculation. For a pair of sites to be tested for LD, the following 3 conditions had to be satisfied to make the calculation meaningful: (i) the 2 sites were at least 2500 bp apart, (ii) there were at least 40 taxa with nonmissing genotypes at both sites being compared, and (iii) at least 2 taxa with minor alleles had to be present at each of the 2 sites.

**Figure 8:**
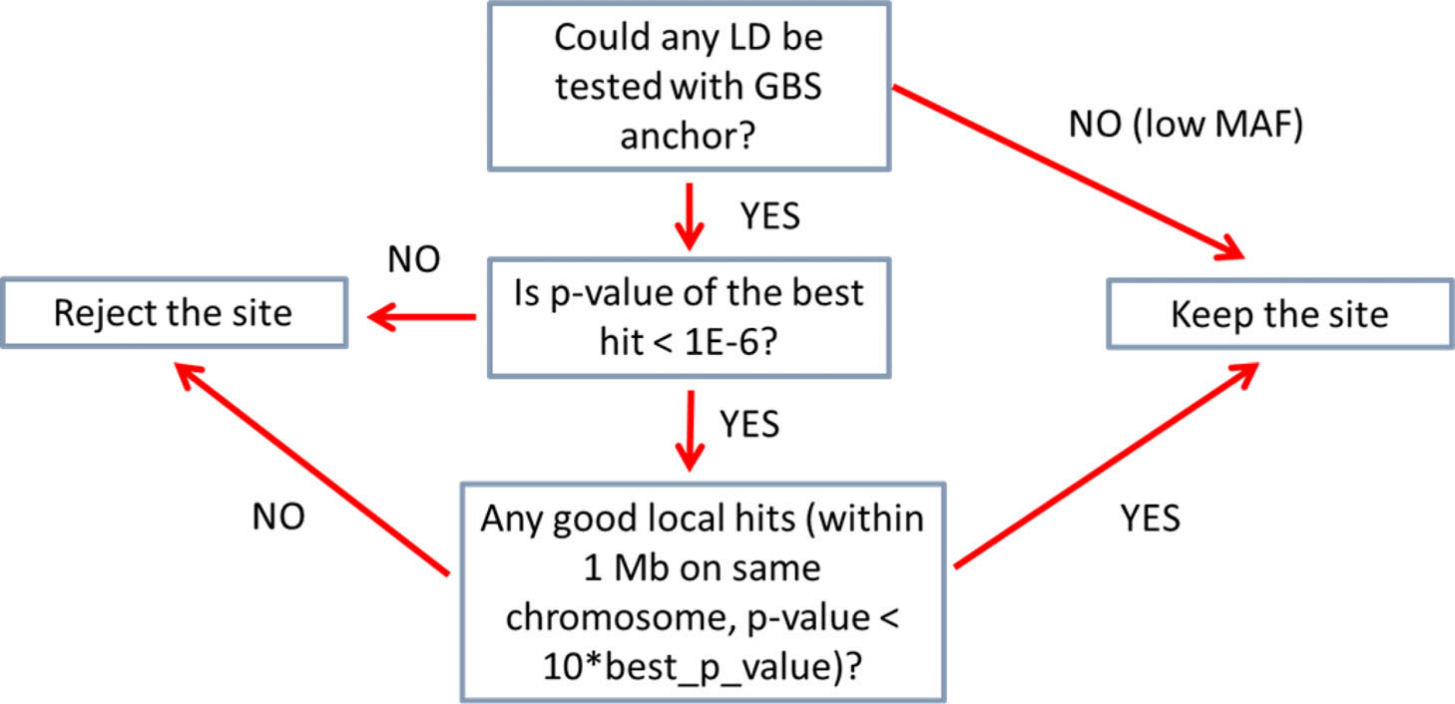
Linkage disequilibrium–based filtering flowchart. The procedure eliminates sites with weak or non-local-only LD hits. Sites with good local LD hits as well as those for which LD could not be probed (because of low MAF) are retained.

The filtering procedure executed for each site is summarized in Fig. 8. First, the LD between the given site and all sites in the GBS anchor was computed, and up to 20 best LD hits (the ones with the lowest *P*-values) were collected. If the *P*-value of the best hit exceeded 1E-6 (which roughly corresponds to the peak of the overall distribution of *P*-values), the site was rejected. Otherwise, it was determined whether the set of best hits contained any local hits, i.e., hits to GBS sites on the same chromosome within 1 Mbp of the site in question and with the *P*-value smaller than 10 times the *P*-value of the best hit. If no such local hits were found, the site was rejected; otherwise it was kept and marked as a site in local LD using the flag “LLD.” Note that the procedure as defined this way filters out sites with only nonlocal LD hits as well as those with only a weak LD signal. Sites in local LD as well as those for which LD could not be assessed (because of low minor allele frequency or missing data) pass the filter.

### Imputation

In the HapMap 3.2.1 pipeline, the ST- and IBD-filtered genotypes, after the application of the additional “>1, >2” depth-based filter, were processed through the LD KNN imputation procedure based on Money et al. [13] to fill in the missing data. The procedure is a version of the “K nearest neighbors” routine where the “nearest neighbors” of a given taxon are selected based on genetic distance computed using variant sites in good local LD. Specifically, for a given target site, a list of up to 70 sites in best LD (as given by the *R*^2^ measure) with it is compiled by checking all surrounding sites within 600 Kb characterized by heterozygosity lower than 3% and more than 50% taxa with nonmissing genotypes. Capping this list at 70 sites leads to good compromise between distance accuracy and computation speed. Then, at the same target site, for each target taxon, up to 30 “nearest neighbor” taxa are selected, with the lowest genetic distances from the target taxon. Genetic distances are computed using the set of local LD sites selected in the previous step. Taxa with more than 50% missing genotypes at LD sites, missing genotype at the target position, having distance from the current taxon larger than 0.1, or resulting in less than 10 common LD sites on which the distance can be calculated, are excluded from the distance calculation process. Genotypes of the selected nearest neighbor taxa at the target site are stored in memory, along with the genetic distances from the target taxon. This information is used to compute a weight *w_i_* of each neighbor genotype *g* as follows:

\begin{equation*}
\ {w_g} = \mathop \sum \limits_i \frac{1}{{1 + 70{d_{gi}}}},\
 \end{equation*}

where the summation index *i* runs over all neighboring taxa with genotype *g* at the target site, and *d_gi_* is the distance of taxon *i* from the target taxon. The genotype with the highest weight is considered the imputed genotype (of the target taxon at the target site), provided its weight is at least 10 times larger than that of the second-best candidate genotype. Otherwise the imputation is considered inconclusive and the imputed genotype is set to “unknown” (missing data), as it is in the case when no close neighbors of the current taxon could be found. If a genotype imputed to “unknown” occurs at a site where MAF <1%, it is automatically converted into a major allele homozygote.

The imputation procedure is run for each genotype in the input file. However, in the output only the originally missing genotypes are updated to imputed ones, whereas all others are left unchanged, even if classified differently. On the other hand, all imputed genotypes are used during a run to collect imputation statistics. The “transition matrix,” showing how many genotypes originally in a given class were imputed into other classes, is an indication of the accuracy of the input genotypes. Error rates calculated from the transition data are given in Table 3.

## Availability of data

At present, reads from all datasets are available via the *GigaScience* repository, *Giga*DB [3] in the form of BAM files (with Illumina sequencing reads aligned to AGP v3 reference) on CYVERSE Data Commons [19], as well as via the NCBI Sequence Read Archive. The 4 datasets used for this project include:

1. dataset “282–×2” and “282–×4”: NCBI BioProject PRJNA389800;
2. dataset “German”: NCBI BioProject PRJNA260788;
3. dataset “hapmap2”: NCBI BioProject PRJNA283986;
4. Dataset “hapmap3.1.1”: NCBI BioProject PRJNA399729.

Datasets 1–4 are also available via Cyverse [19] and include the following:

- the set of HapMap 3.1.1. polymorphisms determined for 916 taxa (from datasets “HapMap2,” “HapMap2 extra,” “CAU,” and “CIMMYT/BGI”) in VCF format;
- the HapMap 3.2.1 variants for 1210 taxa (916 initial Hapmap 3.1.1 taxa + 263 taxa from “282–×2” set + 31 “German” lines) Files c*_282_corrected_onHmp321.vcf.gz in CYVERSE Data Commons;
- unimputed genotypes on HapMap 3.2.1 sites from the full-depth data for the “282” panel (271 taxa, datasets “282–×2” + “282–×4”).

Custom scripts and Java code used in the pipeline are available via bitbucket [16], and an archival copy is also available via *Giga*DB [3].

## Supplementary Material

GIGA-D-17-00007_Original_Submission.pdf

GIGA-D-17-00007_Revision_1.pdf

GIGA-D-17-00007_Revision_2.pdf

GIGA-D-17-00007_Revision_3.pdf

GIGA-D-17-00007_Revision_4.pdf

Response_to_Reviewer_Comments_Original_Submission.doc

Response_to_Reviewer_Comments_Revision_1.doc

Response_to_Reviewer_Comments_Revision_2.doc

Response_to_Reviewer_Comments_Revision_3.doc

Reviewer_1_Report_(Original_Submission) -- Delphine Fleury 10 Apr 2017 Reviewed

Reviewer_2_Report_(Original_Submission) -- Gareth Linsmith 09 May 2017 Reviewed

Additional files

## Additional files

Additional file 1: HapMap3TaxaAndCoverage.xlsx—spreadsheet with a list of all lines used in HapMap 3 with their corresponding coverage.

Additional file 2: DepthFormatDetails.pdf—details of byte representation and storage format used for allelic depths.

## Abbreviations

CIMMYT: International Maize and Wheat Improvement Center; GBS: genotyping-by-sequencing; IDB: identity-by-descent; Indel: insertion-deletion; LD: linkage disequilibrium; LDKNN: linkage disequilibrium-base K nearest neighbor imputation; LLD: sites in local LD; MAF: minor allele frequency; MAPQ: read mapping quality; NAM: nested association mapping; SNP: single nucleotide polymorphism; ST: segregation test; VCF: variant call format.
