# Supplementary material for: Construction of the third-generation Zea mays haplotype map
Source: Gigascience. 2017 Dec 30;7(4):gix134. doi: 10.1093/gigascience/gix134 (PMC5890452; doi:10.1093/gigascience/gix134)
Supplement: Additional files [file gix134_supp.zip › DepthFormatDetails.docx]

**Construction of the third generation *Zea mays* haplotype map**

R. Bukowski *et al.*

Additional file 2

# Byte representation of allelic depths

Most of the per taxon allelic depths encountered in the HapMap 3 datasets are lower than 100. It is thus possible, without significant loss of accuracy, to represent these depths as byte variables rather than integers and therefore save disk space. Depths up to 127 can be represented directly as byte variables, whereas for higher depths one can utilize negative byte values as follows:

$$B=\left\{ \begin{matrix} I & \mathrm{for} I\leq127 \\ 127-I & \mathrm{for} 127<I\leq M \\ \underset{}{Int(max} [-\log_{b} \left( I-o \right),-128]) & \mathrm{for} I>M \end{matrix} \right.$$

The inverse transformation is as follows:

$$I=\left\{ \begin{matrix} B & \mathrm{for} B\geq0 \\ 127-B & \mathrm{for} 0>B\geq127-M \\ \mathrm{Int}(o+b^{\frac{1}{2}-B}) & \mathrm{for} B<127-M \end{matrix} \right.$$

The operator Int() truncates the real value to the smallest integer and the parameters $b$, $o$, and $M$ were chosen as 1.0746, 126, and 182, respectively. With this choice, all the byte values map to unique integers and encoding is exact not only for depths from 0 to 127, but also somewhat beyond (up to $M=182$). Determining $M$ is a bit tricky, as one needs to make sure the corresponding negative byte values won't be also needed in the exponential approximation. For base $b=1.0746$, we found that bytes -1 through -55 can be used directly, allowing exact encoding for depths up to 127+55=182. For depths slightly above 182 the relative error envelope is about 1%, then it grows to 3% for depths around 1,000 to asymptotically reach 3.5% for larger depths (Figure S1). The asymptotic error rate is determined by the base $b$ and equals $(b-1)/2$. The value of $b$ also determines the largest depth that can be effectively approximated. In our case, it is 10,117 approximated as 10,482 (all larger depths are encoded as -128 and then decoded as 10,482). Decreasing the value of $b$ would lead to a better approximation, but smaller maximum representable depth. For example, using 1.05545 allows for depths only up to about 1000, but values up to 210 would be encoded exactly and asymptotic relative error would be 2.5% instead of 3.5%.

# Storage model for allelic depths and average base qualities

The read depths for all six alleles extracted for each taxon and genomic position using samtools mpileup are stored on disk to be retrieved during the joint genotyping step. As shown in Figure S2, the array of allelic depths is usually sparse, especially at low coverage levels, when only one allele (or not at all) is present at each position. To save disk space, the following scheme was used to represent this array within HDF5 structure. Each HDF5 file (one per taxon per chromosome) consists of three byte arrays. Each byte of the first array represent a position on the chromosome (all positions are represented) and shows – through set bits - which alleles have non-zero depths at this position. The second array stores the byte-encoded non-zero allelic depths. The genomic position and the allele represented by each entry of this array is determined by non-zero bits of the first array, as shown in Fig. S2. The average base quality scores are stored in a similar way in the third array.

Effectiveness of this compression scheme depends on the sparsity of the original array and is the highest for taxa with low coverage


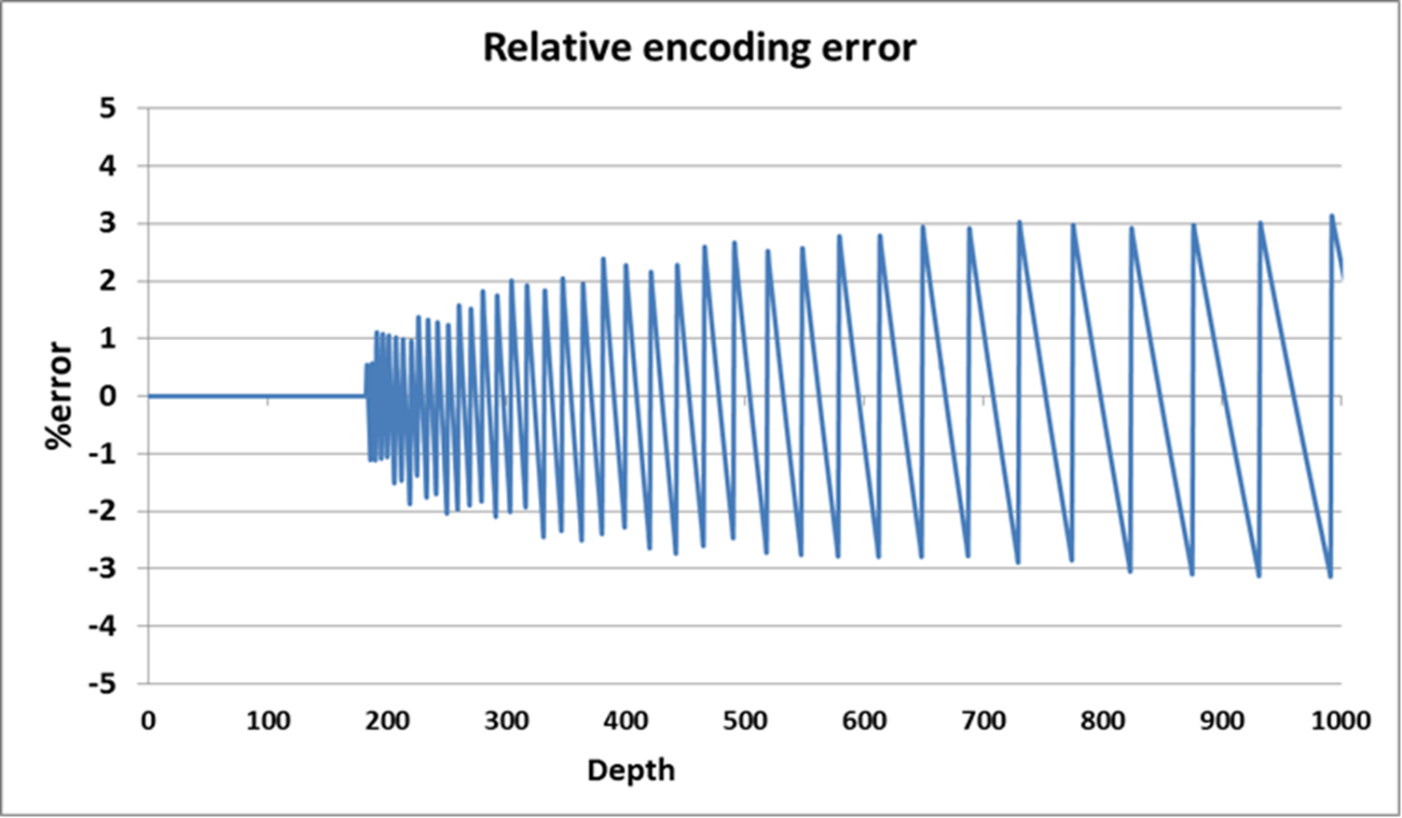


Figure S1: Relative error of depth encoding as byte variables. All values up to 182 are represented exactly. Encoding values 182 through 10,117 and decoding them leads to error of no more than 3.5%. Integers larger than 1 are encoded as -128 and decoded as 10,482.


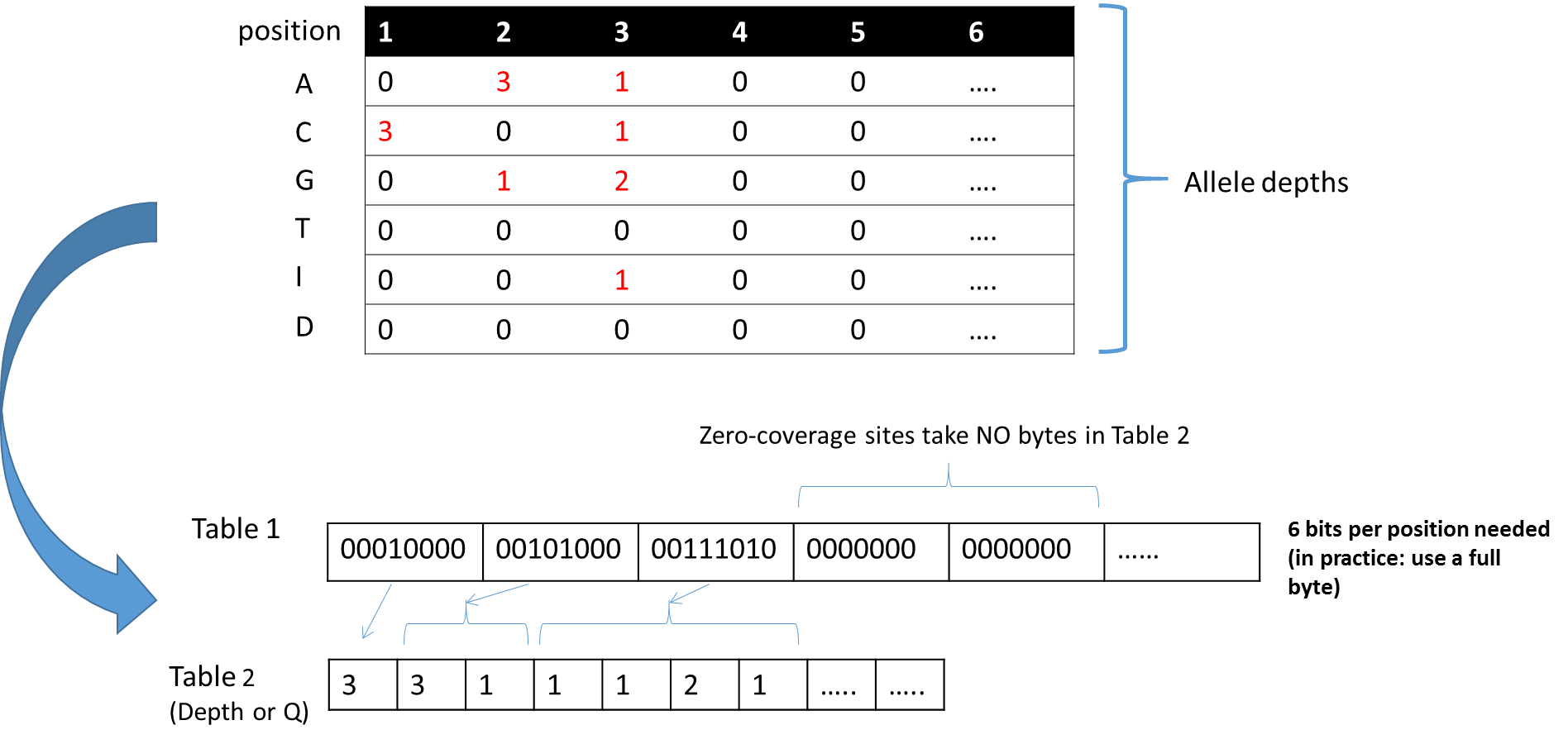


Figure S2: Representing the array of allele depths (or average base qualities) in HDF5 file.
