# Supplementary material for: Construction of the third-generation Zea mays haplotype map
Source: Gigascience. 2017 Dec 30;7(4):gix134. doi: 10.1093/gigascience/gix134 (PMC5890452; doi:10.1093/gigascience/gix134)
Supplement: Response_to_Reviewer_Comments_Original_Submission.doc [file gix134_response_to_reviewer_comments_original_submission.doc]

### Author's Response To Reviewer Comments

Top of Form

Bottom of Form

Dear editor and reviewers,

We would like to thank all editor and reviewers for valuable suggestions. We corrected the typos and addressed minor issues that have been pointed out. Below, we focus on more serious points raised by the reviewers. We hope that with these answers and the modifications we made throughout the manuscript, you will find it suitable for publication in Giga Science.
We added one more author to the list - Anne Lorant of UC Davis. Anne is mostly responsible for generating libraries for the "282" panel and was omitted by mistake.
We are still in the process of uploading the raw data to SRA archive. The "282" panel sequence has already been uploaded with the bioproject accession PRJNA389800 (provided in the manuscript in section Availability of data).
Answers to Reviewer 1 comments:
-p 5 line 53: why did the authors choose 30 as a threshold for MAPQ?
This cut-off was chosen at mid-point between the highest MAPQ value reported by the aligner, corresponding to unambiguous alignments (60) and that of the most ambiguous ones (0). Analysis of the inbreeding coefficient (Section HapMap 3.1.1) and of MAPQ distributions shows that our choice of cut-off leads to decent quality genotypes while allowing for over 80% of alignments with MAPQ>0 to be included. We added this clarification to the text in section ANALYSIS/Initial variant discovery.
-p 5 lines 58-60: the sentence is unclear to me. What is the null hypothesis here? "Sites of high probability" of what?
The null hypothesis is that the observed allelic depths are randomly distributed among taxa. We rewrote the whole fragment about the segregation test in this section to make it more clear. Also, more technical details of the test are presented in section METHODS/Filtering.
-p 7 line 57: why a local realignment wasn't done?
This issue has been addressed in section METHODS/Alignment. Local re-alignment is intended to create consensus alignment in indel regions when read depth is high. It is very intensive computationally and not practical for a project of this scale. Since false variants resulting from incorrect alignments around indels tend to be random, some of such variants are eliminated by the IBD and local LD-based filters. Nevertheless, since the filtering is not perfect, we decided to flag all indels and SNPs with 5 bp of an indel as unreliable ("NI5" flag).
-p 8 line 51: what is the purpose of calculating inbreeding coefficient? The paragraph should start by explaining this.
Inbreeding coefficient was used to estimate genotyping errors, as low inbreed coefficient (high heterozygosity) in inbred lines are mostly due to genotyping errors. The relevant fragment of section ANALYSIS/HapMap 3.1.1 has been re-written.
-p 17 line 31: I find this sentence unclear. By "reads with non-zero mapping quality", do you mean reads with a correct location?
The notion "reads with non-zero mapping quality" was used instead of “reads with a correct location” because there is no way to say for sure whether the reported read location is correct or not. All we know is the (phred-scaled) probability (i.e., mapping quality, denoted as MAPQ), provided by the aligner software (here: bwa mem), that the reported location is incorrect. Thus, the higher MAPQ, the better chance that the read has been placed in correct location.
-p 22 line 46: why did you choose the number of 70 sites in best LD?
As many parameters used in this work, the threshold 70 was chosen by trial and error to provide sufficient accuracy (in this case - of genetic distance calculation) while keeping the computational cost at bay. We added an explanation in this spirit to the relevant fragment of section METHODS/Imputation.
- There are a lot of jargon and acronyms in Figures that make them difficult to read. As most people read the figures first, I suggest you add information in the titles (acronyms and purpose or conclusion).
We expanded the figure captions to make the figures more self-explanatory.

Answers to Editorial Advice comments:
It is important to describe in more detail the criteria chosen, the rationale for the respective thresholds picked, and underlying assumptions made. I felt that at least in some cases, the information provided about those criteria was not sufficient.
We added comments throughout the text addressing the chosen parameters and thresholds. In general, HapMap3 was designed to provide an inclusive set of tentative variants, annotated with various flags and parameters to allow selection of subsets of varying quality. From this point of view, the exact values of various pipeline parameters and thresholds are not essential as long as they are reasonable, which we are confident is the case here.
...it would have been helpful to quantify how this changed the outcomes compared to earlier Hapmapping. For example, the dataset that has been used to generate HapMap 2 could have been reanalyzed with the new pipeline, and differences in outcomes using the new pipeline been pointed out.
The HapMap 3 pipeline described in this paper relies on IBD and LD filters which can be implemented only with large number of taxa. The dataset used in HapMap2 work contained only 104 taxa, about 10 times less than current work. While there were some IBD regions found among these 104 taxa that were used in Ref. [1] as a training set for regression model, it would not be possible to use explicit IBD filtering for each locus, as in the HapMap3 pipeline.
- Page 16, lines 11-12: what is meant by “new sequence marked as originating from line CML103 actually represents material that is significantly more heterozygous from the line with the same name”? It would be useful to show results on how different lines with same name were found to be different
Different members of the consortium contributed sequence datasets described as originating from taxon CML103. However, comparison of genotypes resulting from these different datasets showed significant differences in some parts of the genome. This fragment was re-written for better clarity.

- P 18, l. 39: Provide a reference for the “N+1 problem”
We did not find any formal reference for this otherwise well-known problem. We therefore removed the acronym from the manuscript.
- P 19: I don’t understand the rationale of the segregation test filter. Needs better explanation, why it is justified to use it.
We re-wrote the relevant fragment in section ANALYSIS/Initial variant discovery; more technical details on the ST filter are also given in section METHODS/Filtering. For a population of inbred (i.e., mostly homozygous) lines, read depth corresponding to different alleles at a locus is expected to be concentrated in different subsets of taxa. The ST filter eliminates tentative variant sites where the read depth distribution over taxa is random.
- P20: “At least 200 comparable GBS sites (i.e., non-missing data simultaneously on both lines being compared) were assumed necessary to make the genetic distance calculation feasible.” Why 200 ?
This choice allowed for good distance estimate while keeping the number of detected IBD relationships large.
- P 21: Explain, what you mean by “The raw (ST-filtered) genotypes were checked against the IBD pairs in various regions,etc”
We re-wrote the last paragraph of section METHODS/Filtering/GBS anchor map and IBD filter to present the IBD filtering procedure more clearly.
- P21: Explain “heterozygous genotypes were treated as homozygous in minor allele.”
During the haplotype count calculation of the 2 by 2 contingency matrix used in our LD test, taxa heterozygous at either of the sites being correlated contribute 2 or 4 haplotypes, which tends to somewhat "wash out" the LD signal, and also complicate the calculation. We therefore decided, for the purpose of the LD test, to treat each heterozygous genotype as homozygous in minor allele, which resulted in each taxon contributing only one haplotype.

- Page 6: “At roughly half of the sites surviving this filter, minor allele was not present in IBD contrasts. Such sites, typically with low minor allele frequency, are less reliable and have been marked with “IBD1” flag”. Why are those sites less reliable ? If IBD, my understanding is that regions IBD between lines should not differ. Thus, markers indicating same genotype in these IBD regions should be reliable ?
The essence of the IBD filter, as implemented here, is to compare, at a given locus, genotypes of taxa that have been determined to be in IBD relationships in a region containing that locus. Sometimes, this subset of taxa being tested for IBD does not contain any taxa with minor allele, i.e., the minor allele genotype is not compared to anything during the IBD test. In such a case, even if the test is successful, it does not in any way confirm the minor allele which may still be a false positive. On the other hand, if the minor allele does occur in the subset of taxa tested for IBD, a successful test implies that at least two taxa carry this allele, strengthening the case for its presence.
- Mapping quality needs to be defined somewhere and a reference given. I assume, this refers to the PHRED scale – however, readers should not need to make guesses..
Definition of mapping quality has been added in the first paragraph of section ANALYSIS/Initial variant discovery.
- Figure 4: calculations of inbreeding coefficients depend on assumptions made, which generation was considered unrelated. Spell out, how inbreeding coefficient was calculated, provide reference"
The inbreeding coefficient has been calculated using the VCFtools package. Definition of this quantity has been added in section ANALYSIS/HapMap 3.1.1. Also, the whole paragraph has been changed to emphasize the use of inbreeding coefficient as a probe of genotype quality as a function of mapping quality threshold.

Answer to Reviewer 2 comments:
We looked into Cortex approach, in which variants are called from de Bruijn graph constructed from sequencing reads. Cortex would address the reference genome alignment issues. However, it would now resolve two other problems. 1. Almost all our lines have very low depth of sequencing data with majority of them below 5; 2. Compared with human genomes, maize has very active retro-elements, which results in not just a large number of repeat regions but also very young repeats with little accumulated mutation. De Bruijn graph for maize has been very difficult to resolve outside the genic regions, as indicated by the fact that no single de Bruijn graph-based assembly has ever been published after the first maize genome (from Sanger sequencing), despite of extensive effort. The goal of this project is to identify a set of maize genetic variants that are relative stable in the species and co-linear across the species. Much of the effort of this work was to use population genomics information (IBD, local ID, e.t.c.) to filter out variants in unstable regions of the genome that are not collinear between individuals. We believe the solution is to switch to a pan-genome reference instead of a single reference genome. The version of maize hapmap described in this paper will be the last maize hapmap based on a single reference.

Robert Bukowski, Xiaosen Guo, Yanli Lu, Cheng Zou, Bing He, Zhengqin Rong, Bo Wang, Dawen Xu, Bicheng Yang, Chuanxiao Xie, Longjiang Fan, Shibin Gao, Xun Xu, Gengyun Zhang, Yingrui Li, Yinping Jiao, John Doebley, Jeffrey Ross-Ibarra, Anne Lorant, Vince Buffalo, M. Cinta Romay, Edward S. Buckler, Yunbi Xu, Jinsheng Lai, Doreen Ware, and Qi Sun
