# Supplementary material for: Construction of the third-generation Zea mays haplotype map
Source: Gigascience. 2017 Dec 30;7(4):gix134. doi: 10.1093/gigascience/gix134 (PMC5890452; doi:10.1093/gigascience/gix134)
Supplement: Response_to_Reviewer_Comments_Revision_1.doc [file gix134_response_to_reviewer_comments_revision_1.doc]

### Author's Response To Reviewer Comments

Top of Form

Bottom of Form

In this revised version, the reference to the supporting data has been added, and new NCBI accessions have been added for each data set.
