# Supplementary material for: Construction of the third-generation Zea mays haplotype map
Source: Gigascience. 2017 Dec 30;7(4):gix134. doi: 10.1093/gigascience/gix134 (PMC5890452; doi:10.1093/gigascience/gix134)
Supplement: Response_to_Reviewer_Comments_Revision_2.doc [file gix134_response_to_reviewer_comments_revision_2.doc]

### Author's Response To Reviewer Comments

Top of Form

Bottom of Form

Revised as advised, including reference to GigaDB, using CyVerse URL instead of file path, and reducing corresponding authors to 2. The CyVerse file paths were attached to the GigaDB database.
