# Supplementary material for: Construction of the third-generation Zea mays haplotype map
Source: Gigascience. 2017 Dec 30;7(4):gix134. doi: 10.1093/gigascience/gix134 (PMC5890452; doi:10.1093/gigascience/gix134)
Supplement: Response_to_Reviewer_Comments_Revision_3.doc [file gix134_response_to_reviewer_comments_revision_3.doc]

### Author's Response To Reviewer Comments

Top of Form

Bottom of Form

Bitbucket Link to get pipelines is added.
