# Supplementary material for: Construction of the third-generation Zea mays haplotype map
Source: Gigascience. 2017 Dec 30;7(4):gix134. doi: 10.1093/gigascience/gix134 (PMC5890452; doi:10.1093/gigascience/gix134)
Supplement: Reviewer_1_Report_(Original_Submission) -- Delphine Fleury — 10 Apr 2017 Reviewed [file gix134_reviewer_1_report_(original_submission).docx]

**Reviewer Report**

**Title:** Construction of the third generation Zea mays haplotype map

**Version:** Original Submission	**Date:** 10 Apr 2017

**Reviewer name:** Delphine Fleury

**Reviewer Comments to Author:**

The manuscript describes the dataset and bioinformatics pipeline enabling the construction of a haplotype map based on sequences of ~1,200 maize accessions. Haplotype map developed from thousands of accessions is a key resource of information for maize genetic and breeding. Beyond the importance of this work for the maize community, the pipeline describes here will be very useful for other crops with similar issues than maize, including a large genome with a high number of duplicated regions, gene copies and repetitive elements.
Overall, the manuscript is well written with adequate level of information provided. The work described here is scientifically sound and explained logically. Although it's the third generation of maize haplotypes map, the manuscript includes enough novelty to justify a publication, with the improvement of the pipeline for minor allele calls and heterozygous calls. The use of flag in the VCF files indicating the characteristics of the variant sites will also help the readers to appropriately use the data. The data support the conclusion p 15 which is an important message for the readers.

I have some minor corrections and a couple of points that need clarification:
-p 3 line 16: add the acronyms CAU for China agricultural university, as used later in the text
-add a space between numbers and bp unit all through the text
-Tables 1 and 2 are difficult to read. It would be easier to separate the results in Table 1 under "Coverage per taxon" in different columns instead of separating them by a coma. Please separate 3.1.1 from 3.2.1unimp and 3.2.1imp in different columns in Tables 1 and 2.
-There a few typo such as CIMYYT page 5 line 46.
-p 5: the acronyms should be described in the text: base quality (BQ) in line 51, mapping quality (MAPQ) in line 53.
-p 5 line 53: why did the authors choose 30 as a threshold for MAPQ?
-p 5 lines 58-60: the sentence is unclear to me. What is the null hypothesis here? "Sites of high probability" of what?
-p 7 line 25: delete the dot point at the beginning of the sentence.
-p 7 line 57: why a local realignment wasn't done?
-p 8 line 51: what is the purpose of calculating inbreeding coefficient? The paragraph should start by explaining this.
-p 8 line 54: Is lower threshold q1? If so, please spell it out.
-p 9 lines 7-9: This sentence explains the aim of Figure 4 and should be at the beginning of the paragraph.
-p 10 line: please spell out the acronym DUP. Since M&M is at the end, it's best to assume that the reader hasn't read it yet.
-p 11 line 41: spelling mistakes (failed the LD filter?)
-p 13 lines 26 and 29: replace coma by semi-colon to clearly separate the % results.
-p 14 line 14: "to capture real signal related to phenotypic expression". I find this expression a bit odd. Do you mean "to capture a true association with phenotype"?
-p 15 line 31: replace coma by colon in (teosinte lines: 17 Z. mays…)
-p 15 line 51: spell out the acronym (North Central Regional Plant Introduction Station)
-p 16 lines 4-21: this paragraph doesn't match the writing quality of the rest of the paper as if it wasn't written by the same author or hasn't been reviewed. The writing is unnecessary complicated and a bit confusing for the reader.
-p 16 line 32: replace 113.702 billions by 113.7 billions for ease to read; add "were obtained on 1,218 taxa".
-p 16 line 44: replace better by higher
-p 17 line 31: I find this sentence unclear. By "reads with non-zero mapping quality", do you mean reads with a correct location?
-p 21 lines 20-39: I find this paragraph difficult to understand.
-p 22 line 46: why did you choose the number of 70 sites in best LD?
-p 22 line 24: replace coma by semi-colon
-p 22 line 58: "taxa with less than 50% non-missing genotypes". It would be simpler to say taxa with more than 50% missing genotypes.
-p 23 line 11: delete the in "this information is the used to compute"
-p 25 lines 7-12: Some acronyms are missing: AGP, MAPQ, BQ, LDKNN, NI5, LLD, NO, DUP, VCF.
- There are a lot of jargon and acronyms in Figures that make them difficult to read. As most people read the figures first, I suggest you add information in thee titles (acronyms and purpose or conclusion).

**Methods**

Are the methods appropriate to the aims of the study, are they well described, and are necessary controls included? Yes

**Conclusions**

Are the conclusions adequately supported by the data shown? Yes

**Reporting Standards**

Does the manuscript adhere to the journal’s guidelines on [minimum standards of reporting?](https://academic.oup.com/gigascience/pages/Minimum_Standards_of_Reporting_Checklist) Yes

**Statistics**

Are you able to assess all statistics in the manuscript, including the appropriateness of statistical tests used? No, I do not feel adequately qualified to assess the statistics.

**Quality of Written English**

Please indicate the quality of language in the manuscript: Acceptable

**Declaration of Competing Interests**

Please complete a declaration of competing interests, considering the following questions:

- Have you in the past five years received reimbursements, fees, funding, or salary from an organisation that may in any way gain or lose financially from the publication of this manuscript, either now or in the future?
- Do you hold any stocks or shares in an organisation that may in any way gain or lose financially from the publication of this manuscript, either now or in the future?
- Do you hold or are you currently applying for any patents relating to the content of the manuscript?
- Have you received reimbursements, fees, funding, or salary from an organization that holds or has applied for patents relating to the content of the manuscript?
- Do you have any other financial competing interests?
- Do you have any non-financial competing interests in relation to this paper?

If you can answer no to all of the above, write 'I declare that I have no competing interests' below. If your reply is yes to any, please give details below.

I declare that I have no competing interests.

I agree to the open peer review policy of the journal. I understand that my name will be included on my report to the authors and, if the manuscript is accepted for publication, my named report including any attachments I upload will be posted on the website along with the authors' responses. I agree for my report to be made available under an Open Access Creative Commons CC-BY license (http://creativecommons.org/licenses/by/4.0/). I understand that any comments which I do not wish to be included in my named report can be included as confidential comments to the editors, which will not be published.

I agree to the open peer review policy of the journal

To further support our reviewers, we have joined with Publons, where you can gain additional credit to further highlight your hard work (see: https://publons.com/journal/530/gigascience). On publication of this paper, your review will be automatically added to Publons, you can then choose whether or not to claim your Publons credit. I understand this statement.

Yes
