# Supplementary material for: Construction of the third-generation Zea mays haplotype map
Source: Gigascience. 2017 Dec 30;7(4):gix134. doi: 10.1093/gigascience/gix134 (PMC5890452; doi:10.1093/gigascience/gix134)
Supplement: Reviewer_2_Report_(Original_Submission) -- Gareth Linsmith — 09 May 2017 Reviewed [file gix134_reviewer_2_report_(original_submission).docx]

**Reviewer Report**

**Title:** Construction of the third generation Zea mays haplotype map

**Version:** Original Submission	**Date:** 09 May 2017

**Reviewer name:** Gareth Linsmith

**Reviewer Comments to Author:**

Due to the difficulties you discuss with calling variants against a single reference, perhaps reference free SNP calling (for example cortex var) is worth a try on your high coverage samples only (not an option for low coverage samples of course).

**Methods**

Are the methods appropriate to the aims of the study, are they well described, and are necessary controls included? Yes

**Conclusions**

Are the conclusions adequately supported by the data shown? Yes

**Reporting Standards**

Does the manuscript adhere to the journal’s guidelines on [minimum standards of reporting?](https://academic.oup.com/gigascience/pages/Minimum_Standards_of_Reporting_Checklist) Yes

**Statistics**

Are you able to assess all statistics in the manuscript, including the appropriateness of statistical tests used? No, I do not feel adequately qualified to assess the statistics.

**Quality of Written English**

Please indicate the quality of language in the manuscript: Acceptable

**Declaration of Competing Interests**

Please complete a declaration of competing interests, considering the following questions:

- Have you in the past five years received reimbursements, fees, funding, or salary from an organisation that may in any way gain or lose financially from the publication of this manuscript, either now or in the future?
- Do you hold any stocks or shares in an organisation that may in any way gain or lose financially from the publication of this manuscript, either now or in the future?
- Do you hold or are you currently applying for any patents relating to the content of the manuscript?
- Have you received reimbursements, fees, funding, or salary from an organization that holds or has applied for patents relating to the content of the manuscript?
- Do you have any other financial competing interests?
- Do you have any non-financial competing interests in relation to this paper?

If you can answer no to all of the above, write 'I declare that I have no competing interests' below. If your reply is yes to any, please give details below.

I declare that I have no competing interests.

I agree to the open peer review policy of the journal. I understand that my name will be included on my report to the authors and, if the manuscript is accepted for publication, my named report including any attachments I upload will be posted on the website along with the authors' responses. I agree for my report to be made available under an Open Access Creative Commons CC-BY license (http://creativecommons.org/licenses/by/4.0/). I understand that any comments which I do not wish to be included in my named report can be included as confidential comments to the editors, which will not be published.

I agree to the open peer review policy of the journal

To further support our reviewers, we have joined with Publons, where you can gain additional credit to further highlight your hard work (see: https://publons.com/journal/530/gigascience). On publication of this paper, your review will be automatically added to Publons, you can then choose whether or not to claim your Publons credit. I understand this statement.

Yes
